# High-Level Synthesis of Online K-Means Clustering Hardware for a Real-Time Image Processing Pipeline

**DOI:** 10.3390/jimaging5030038

**Published:** 2019-03-14

**Authors:** Aiman Badawi, Muhammad Bilal

**Affiliations:** Electrical and Computer Engineering Department, King Abdulaziz University, Jeddah 21589, Saudi Arabia

**Keywords:** image segmentation, K-Means, image processing pipeline, FPGA, high-level synthesis

## Abstract

The growing need for smart surveillance solutions requires that modern video capturing devices to be equipped with advance features, such as object detection, scene characterization, and event detection, etc. Image segmentation into various connected regions is a vital pre-processing step in these and other advanced computer vision algorithms. Thus, the inclusion of a hardware accelerator for this task in the conventional image processing pipeline inevitably reduces the workload for more advanced operations downstream. Moreover, design entry by using high-level synthesis tools is gaining popularity for the facilitation of system development under a rapid prototyping paradigm. To address these design requirements, we have developed a hardware accelerator for image segmentation, based on an online K-Means algorithm using a Simulink high-level synthesis tool. The developed hardware uses a standard pixel streaming protocol, and it can be readily inserted into any image processing pipeline as an Intellectual Property (IP) core on a Field Programmable Gate Array (FPGA). Furthermore, the proposed design reduces the hardware complexity of the conventional architectures by employing a weighted instead of a moving average to update the clusters. Experimental evidence has also been provided to demonstrate that the proposed weighted average-based approach yields better results than the conventional moving average on test video sequences. The synthesized hardware has been tested in real-time environment to process Full HD video at 26.5 fps, while the estimated dynamic power consumption is less than 90 mW on the Xilinx Zynq-7000 SOC.

## 1. Introduction

The inclusion of advanced frame analysis techniques in live video streams has now become mandatory in modern smart surveillance systems. Thus, the conventional image processing pipeline of video cameras has transformed in the recent years to include some form of object, scene, and/or event analysis mechanism as well [1]. Strict real-time and minimal power consumption constraints, however, limit the number and the complexity of operations that can be included within the camera modules [2]. Thus, some pre-processing tasks, such as motion estimation, image segmentation, and trivial object detection tasks have attracted the attention of contemporary researchers [3]. Furthermore, the increasing complexity of computer vision systems has led designers to resort to higher-level programming and synthesis tools, to shorten the design time. In this regard, Xilinx High-Level Synthesis (HLS) [4] and Simulink Hardware Description Language (HDL) Coder [5] are two widely cited tools. The latter is particularly suitable for the design of large computer vision systems, since it incorporates extensive functional verification and the ability to compare with built-in standard algorithms. Thus, the HDL coder supports quick synthesis and the functional verification of a large number of image processing algorithms, such as custom filtering, colorspace conversion and image statistics collection, etc. However, the current toolbox version lacks the explicit support for image segmentation tasks. To this end, we have developed a Simulink model to extend the capability of this toolbox to support this vital function. Although, various advance algorithms for scene segmentation have been put forward by researchers in recent years [6,7], we have chosen “Online K-Means” [8,9] to be incorporated in our proposed hardware, to keep logic resource utilization at minimum. Furthermore, it has been demonstrated that the use of weighted averaging in the place of moving averaging leads to a reduction in logic resource requirements, without compromising the result precision. Thus, the contributions of the conducted work can be summarized as follows:

- Development of a synthesizable Simulink model for the K-Means clustering operation, which is currently not available as an intrinsic block in the Simulink HDL Coder/Vision HDL Coder toolbox (Matlab R2018b)
- Logic resource conservation through the use of the weighted average in the place of the moving average, which requires costly division operation
- Provision of experimental evidence to demonstrate the utility of the weighted average in preserving the result fidelity of the on-line K-Means algorithm for image segmentation

The proposed design can be downloaded (https://sites.google.com/view/4mbilal/home/rnd/image-segmentation, see Supplementary Materials) as an open-source HDL IP core for its direct incorporation into the image processing pipeline hardware on Xilinx FPGAs. The associated Simulink model and the testing environment are also available for practitioners and researchers, to facilitate further development.

The rest of the paper is organized as follows. Section 2 contains the necessary background, and it discusses the relevant works reported in the literature. Section 3 describes the details of the hardware implementation of the online K-Means algorithm for scene segmentation, using the Simulink HDL Coder toolbox. Section 4 presents the FPGA synthesis and implementation results, as well as a comparison with contemporary works. The discussion is concluded with the identification of possible future directions.

## 2. Background and Literature Review

**Figure 1:**
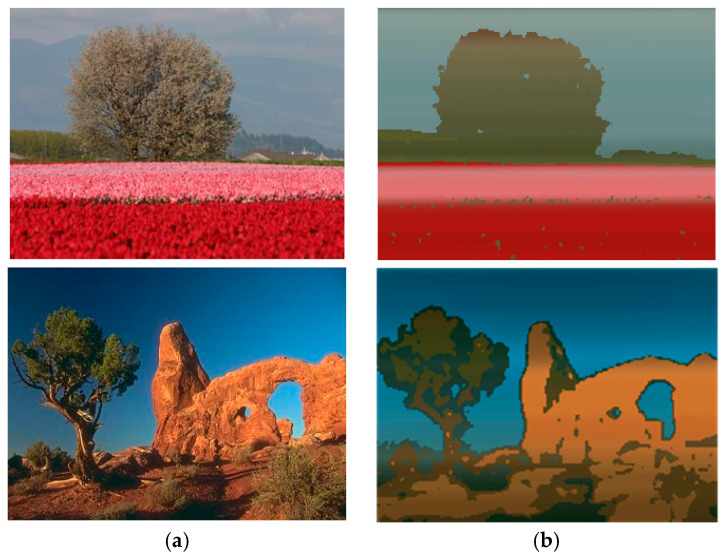
Image segmentation examples: (**a**) Input image [18]; (**b**) Segmented image with each pixel classified as one of the four best matching dominant color clusters (prominent objects) in the input image.

Image or scene segmentation refers to the classification/grouping of pixels, such that each class/group represents a differently perceived object. For this purpose, different features are employed to discriminate one object from another. Texture, boundary, edges, and color are some of the most widely employed features to distinguish distinct objects [6,7,10]. The corresponding numeric representation of these features themselves are obtained through various arithmetic operations, such as gradient filtering, colorspace conversion, and local histogram population [7,11,12,13] etc. The extracted features are then “clustered” to form groups of pixels that are perceived to belong to the same objects. Various clustering algorithms, such as Gaussian Mixture Modelling (GMM) [12,14], Expectation-Maximization (EM) [11,13], K-Means [15,16], and their derivative algorithms [17] have been used by different studies reported in the literature. Some form of post-processing operations, such as ‘region growing’, are also required to assign unclassified pixels or outliers to form a neat and closed boundary around the finally perceived objects. Figure 1 depicts an example of color-based segmentation using a K-Means clustering algorithm without any post-processing.

As mentioned earlier, the inclusion of the image segmentation option as a hardware module inside the image processing pipeline of a camera is constrained by its low-power and complexity requirements. Benetti et al. [19] have recently described the design of an ultra-low-power vision chip for video surveillance, which can detect motion as well as segment the significant portions of the input frames in real-time. This design is limited to specific scenarios with rigid hardware requirements. Moreover, the camera sensor is severely limited in spatial resolution, and is hence, unsuitable for general-purpose applications. Lie et al. [20] have described another neural network-based design for medical imaging applications. Another hardware architecture proposed by Genovese and Napoli [21] uses GMM-based segmentation to extract the foreground (moving objects) from the background. Liu et al. [22] have proposed support vector machine-based image segmentation hardware. These designs target specific applications (e.g., medical imaging and surveillance, etc.), and they are not tailored for inclusion in general-purpose cameras. For general-purpose applications, simpler pixel-based operations are generally preferred over a window-based operation, to reduce the memory and associated power consumption requirements. Color-based segmentation satisfies this requirement, and thus, it naturally stands out favorably over other options, which inevitably require line memory buffers for their operation. Despite being algorithmically simple, color-based segmentation yields promising results, and it has been the subject of various research efforts reported in the literature. Furthermore, since pixel data are presented to the processing hardware in the raster scan order (stream), ‘online’ cluster update algorithms are required. Liang and Klein [23] have demonstrated that ‘online EM’-based clustering in fact performs better than batch processing. Liberty et al. [24] have demonstrated similar results for ‘online K-Means’ algorithm. The latter is more suitable for hardware implementation, since it involves fewer computations, involving fixed-point arithmetic.

Hussain et al. [25] have described an FPGA architecture of a K-Means clustering algorithm for a bioinformatics application to process large genome datasets. Similarly, Kutty et al. [26] have described a fully pipelined hardware for the K-Means algorithm that is capable of running at 400 MHz on a Xilinx target FPGA. These designs, however, lack the ability to classify the incoming data (pixels) online. Thus, these designs necessarily require full-frame storage in the external memory for classification at a later stage. Moreover, the latter work fails to describe how the problem of the inherent feedback loop in the K-Means algorithm has been handled while aggressively pipelining the hardware. Thus, although the attainment of higher speed has been mentioned as a result of the simple insertion of pipeline registers in the distance calculation module, the cluster update feedback loop has been ignored in the overall speed calculation. Recently, Raghavan and Perera [27] have proposed another FPGA-based design for big-data applications. This design also involves frequent memory accesses, and is hence, not suitable for insertion into image processing pipeline. Cahnilho et al. [28] have described a hardware-software co-design approach to implement the clustering algorithm. The involvement of the processor in the operation necessarily complicates the data flow while processing the pixel stream, and is hence, not desirable in real-time systems. Li et al. [29] have used the Xilinx HLS tool to implement AXI4 bus compliant K-Means hardware accelerator. However, this design also uses main memory for the cluster update feedback loop, and it is not suitable for its incorporation in a camera module as a low-complexity add-on. Khawaja et al. [30] have described a multiprocessor architecture to accelerate the K-Means algorithm. This design is meant for parallel processing at several nodes, and it is hence, not suitable for insertion in a real-time image processing pipeline.

It can be noticed from the description of these hardware designs reported earlier in the literature that the color-based online K-Means clustering is a popular choice among researchers, due to its simpler architecture and performance. However, all of these designs allocate a large amount of logic resources for the centroid update mechanism, due to the presence of a divider inside this module. In this work, we propose to circumvent this huge cost by employing a weighted average instead of a moving average for the cluster update. Weighted averaging replaces an explicit division operation with multiplication by constants, and hence, it reduces circuit complexity. This mechanism relies on the temporal redundancy in pixel values of adjacent video frames, and has been shown to work without noticeable loss in accuracy. Moreover, the proposed design is implemented by using high-level synthesis tools (Simulink) for quick insertion into larger systems, and it has been made publicly available as a downloadable FPGA IP core.

## 3. Online K-Means Clustering Hardware Design Using Simulink

The proposed image segmentation hardware accelerator uses an online K-Means clustering algorithm, and it has been designed with a standard Xilinx AXI4 streaming interface, so that it can be inserted as an FPGA IP core within any image processing pipeline flexibly. This section gives a brief overview of the underlying algorithm with some desired modifications, to minimize the hardware resource requirements. This is followed by a detailed description of the proposed hardware architecture.

### 3.1. The Online K-Means Algorithm for Color-Based Image Segmentation

The Online K-Means clustering algorithm is listed as follows.

**Algorithm 1:** Online K-Means clustering algorithm for color-based image segmentation1:Initialize the ‘k’ number of centroids, $C_{1}$, $C_{2}$, $C_{3}....C_{k}$ with random values.2:Initialize the counts n_1_, n_2_, n_3_ .... n_k_ to zero.3:**while** ‘pixel stream continues’ **do**4: p ← RGB2YCbCr(p)5: Match the input pixel, ‘p’, to a single centroid $C_{i}$ by minimizing the distance $\|p-C_{i}{\|}^{2}$
6: Increment n_i_7: Update the matching centroid, $C_{i}$, using moving average8: $\hat{C_{i}}$ ← $C_{i}$ + (1/ n_i_)(p − $C_{i}$)9: Classify the input pixel, ‘p’, as ‘i’.10:
**end**


In our work, we have fixed the number of clusters, ‘*k*’, to be eight. The RGB format for pixel representation is quite commonly used by frame capture and display devices. This representation has been, however, found to be less favorable for color matching in various studies [31,32,33]. The reason for this is that RGB does not yield a perceptually uniform result when different colors are characterized, based on a numeric distance. For this purpose, various researchers have suggested that RGB be converted to LUV or LAB colorspaces [34,35], which yield a much better response (perceptually uniform) to Euclidean distance when differentiating colors. These colorspaces achieve this by decoupling the luminance (illumination) from the color (hue) information, using complex floating-point operations. In our experiments, we have used the YCbCr format, which works similar to LUV and LAB in decoupling the illumination from color information, but it is not as perceptually uniform. The advantage of this, however, is that it is commonly employed by many commercial cameras and almost all compression schemes. Moreover, it can be computed from the intrinsic RGB space by using simpler arithmetic operations as follows:

(1) $$\left[\begin{array}{c} Y\\ C_{b}\\ C_{r} \end{array}\right]=\left[\begin{array}{ccc} 0.299 & 0.587 & 0.114\\ -0.169 & -0.331 & 0.500\\ 0.500 & -0.419 & -0.081 \end{array}\right]\left[\begin{array}{c} R\\ G\\ B \end{array}\right]$$

**Figure 2:**
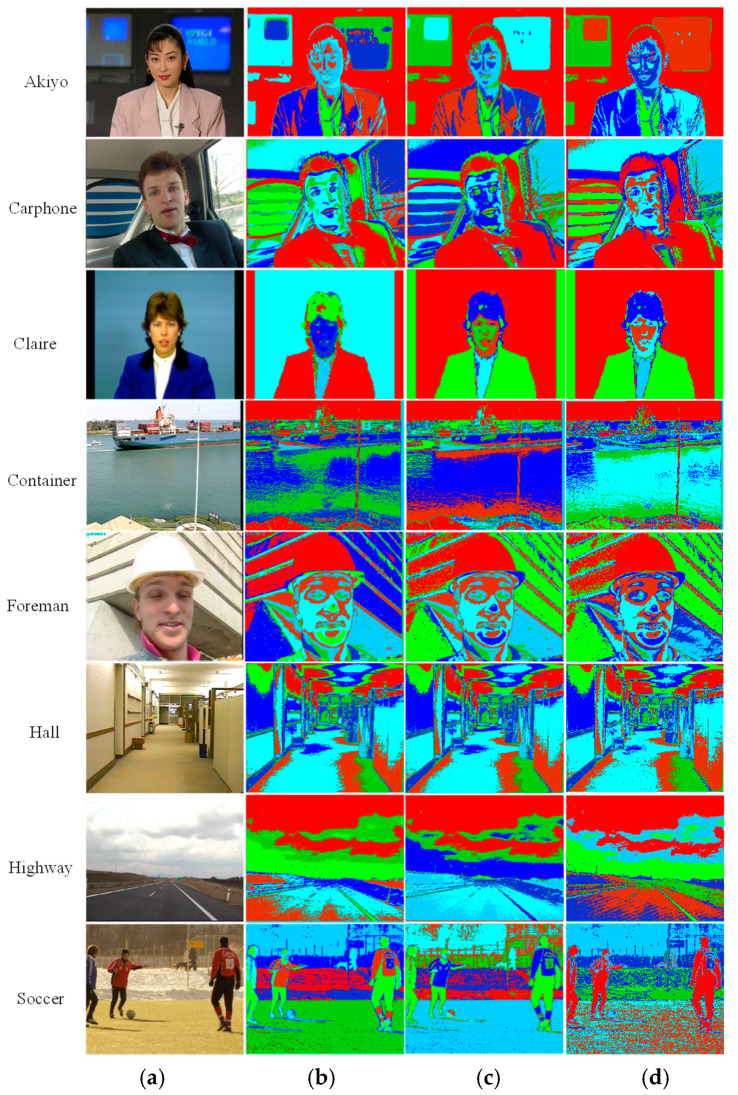
Color-based segmentation using the Matlab intrinsic k-means function: (**a**) Input image; (**b**) Output using the “LAB” colorspace; (**c**) Output using the “YCbCr” colorspace; (**d**) Output using the “RGB” colorspace.

Thus, complex colorspace conversion operations can be entirely skipped if the incoming video stream is already in this format. Figure 2 compares the results of using LAB, YCbCr, and RGB colorspaces for segmentation with the Matlab intrinsic k-means function (L2-norm) on test images. Eight clusters are considered in each case, and they have been depicted by using eight corresponding pseudo-colors. It can be noticed that both LAB and YCbCr colorspaces give visually comparable results. The difference is perceptively discernable only in ‘Akiyo’ and ‘Container’. In fact, in these two cases, YCbCr gives better clustering of the blue screen (Akiyo) and the ocean (Container) than LAB. Prasetyo et al. [36] and Shaik et al. [37] have also noted the utility of the YCbCr colorspace in segmentation operation. Sajid et al. [38] have similarly employed YCbCr for background–foreground clustering. Figure 2 shows that the RGB colorspace works well in the case of pixel groups with markedly different shades of hue and illumination values. However, it fails to account for subtle changes in the illumination values of the pixels belonging to the same object (i.e., similar hue information) and clusters these separately. Thus, the thin outline of the screen in the background of ‘Akiyo’ is wrongly identified as a different object. Similarly, the field is not clustered properly in ‘Soccer’. Both YCbCr and LAB yield a better clustering solution in these cases.

**Figure 3:**
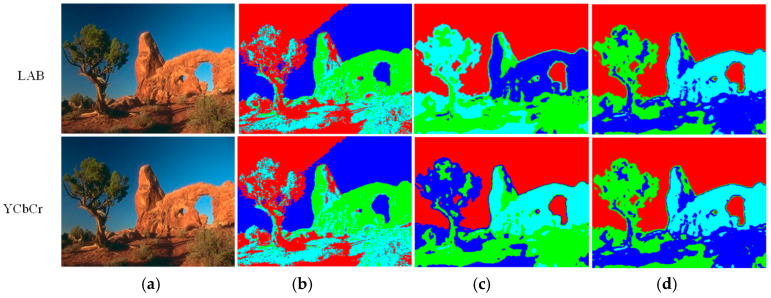
The Effect of using the luminance channel and the distance measure on clustering performance: (**a**) Original image; (**b**) Including the Y/L channel with L2 norm; (**c**) Excluding the Y/L channel with L2 norm; (**d**) Excluding the Y/L channel with L1 norm.

After colorspace conversion, the luminance (intensity) is depicted by the “Y” channel, while chrominance (color information) is described by the other two components, i.e., “Cb” and “Cr”. All three channels can be used to compute the vector distance of the current pixels from the centroids of the respective clusters. However, omitting the luminance channel (Y/L) while computing the distance has favorable results in some cases, as shown in Figure 3. It can be observed that including the luminance information leads to an incorrect segmentation of the sky into two segments, due to the brightness variation (Y/L channel). Removing this channel from the distance calculation rectifies the situation for both the YCbCr and LAB colorspaces. Moreover, using L1-norm in place of L2-norm for distance calculation gives almost identical results. This finding is in line with the extensive experimental results reported by Estlick et al. [39]. They found L1-norm to not only reduce the computational complexity, but also to improve the segmentation results in some cases. In our hardware, the use of L1 or L2-norm and the inclusion/exclusion of the “Y” channel can be selected via independent switches under software control, to facilitate catering to different environments.

In offline applications, the centroids are determined after processing all of the pixels in the given image/frame. The output classification is calculated during the second pass, once all of the centroids are available. In real-time applications, on the other hand, the centroids of the matched cluster (minimum distance) are updated by using the moving average formula. This involves a division operation, and it is the source of major complexity in hardware implementations, as discussed in the previous section. The pixel classification, the matching cluster’s index, ‘*I*’, is simultaneously output.

In order to remove the division operation from the algorithm, we have incorporated the “weighted average” instead of the moving average in step 8 of Algorithm 1. This can be rewritten as:

(2) $$\hat{C_{i}}=C_{i}\left(\frac{n_{i}-1}{n_{i}}\right)+p\left(\frac{1}{n_{i}}\right)$$

The weighted average formula, on the other hand, yields the following formulation:

(3) $$\hat{C_{i}}=C_{i}\left(\propto \right)+p\left(1-\propto \right)$$

where ‘∝’ is a predetermined constant that is close to 1, e.g., ≈0.999. It can be noticed that although the weighted averaging does not involve division, it approximates the moving average in the limit:

(4) $$\underset{n\to \infty }{\lim}\left(\frac{n_{i}-1}{n_{i}}\right)\approx 1$$

**Figure 4:**
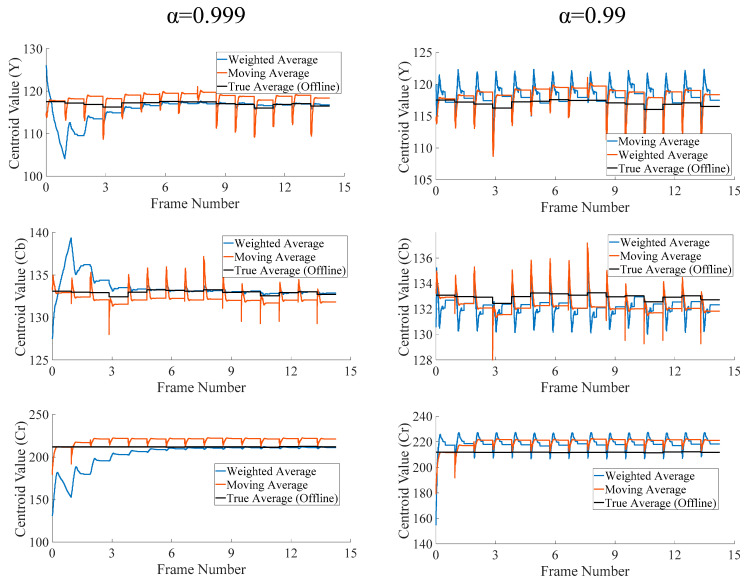
Effect of choice of ‘α’ on a representative centroid’s values for the first 15 frames of ‘Hall’ test video sequence.

Practically, this limit is reached before processing even 10 lines of pixels in a moderate-resolution video frame, such as VGA (640 × 480). Thus, the revised formulation of the averaging operation in Equation (3) removes the need for expensive division operation. This alteration, however, does not affect the clustering performance of the overall algorithm noticeably, since the cluster centroids invariably depict similar variations during the processing of the whole frame, for both the moving and weighted average operations in the online clustering methodology. This behavior has been depicted for a representative centroid during the first 15 frames of the test video sequence “Hall” in Figure 4. It can be observed that both the moving and the weighted averages fluctuate during the processing of the frame, as new pixels are processed in the raster scan order. For reference, centroid values from an offline implementation of K-Means (Matlab intrinsic function) have also been plotted alongside. These have been labelled “True Average”, since offline methods access whole frames at a time to determine the centroid values. These a-priori values remain constant during the second pass of the offline algorithm when pixels are classified. We have plotted these values as references to judge the performance of the moving and weighted average-based on-line methods, respectively. Both the moving and weighted average-based methods initialize the cluster centroids with identical values (seeds) at the start of the first frame. It was noticed that for ∝ = 0.999, the moving average tracks the static true value very well. However, at the start, it takes roughly six frames for all three values (YCbCr) to settle. This “settling” time will be needed whenever rapid scene changes occur in the video frames, and the centroids shift positions. At 15 frames per seconds (fps), this translates to less than half a second. A higher value will further increase this delay. Decreasing ∝ to 0.99, decreases the settling time to just one frame but also leads to more fluctuation. It is worth noting that even at this rate, it causes lower fluctuations than the moving average. Thus, the weighted average is a better choice in either case.

**Figure 5:**
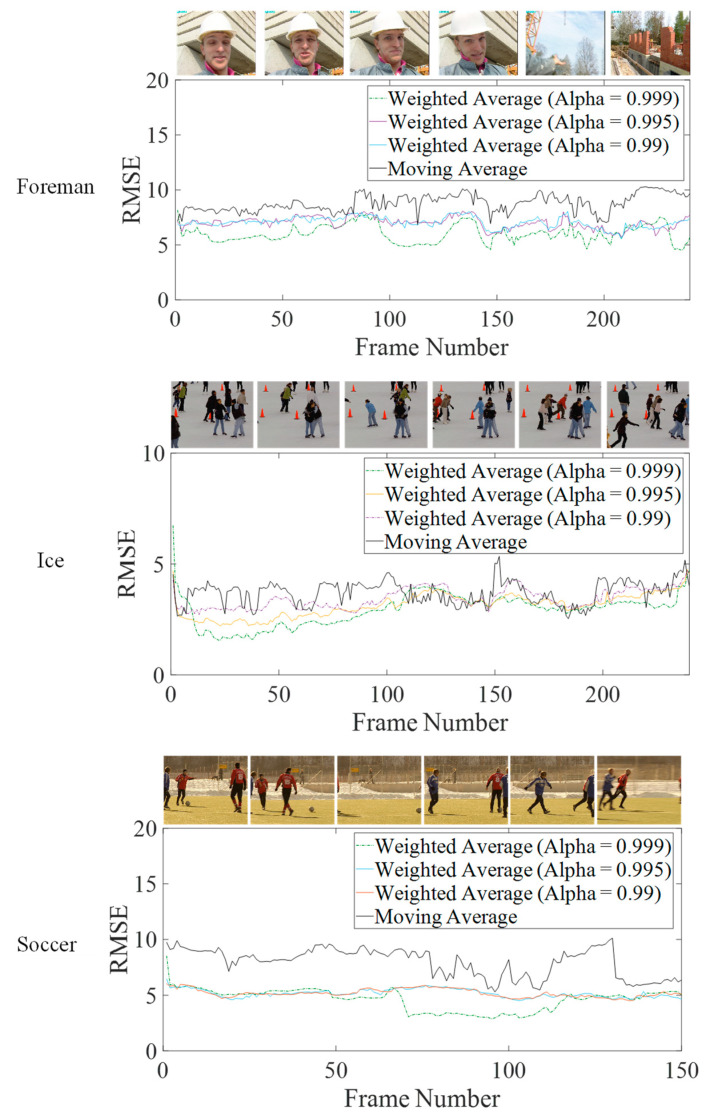
Error deviation in the centroid of the online K-Means algorithms with respect to reference offline algorithm on test video sequences with high motion content.

To further investigate the performance of weightage average-based on-line algorithm, the combined error in the calculation of all of the centroid centers with reference to standard offline implementation has been gathered on test video sequences. The root means squared error (RMSE) has been used as the metric to evaluate different settings, and it has been reported on per-frame basis. Eight clusters have been considered in all of the experiments. “Y” information is included and L2-norm is used for distance calculation. The centroids are randomly initialized around the middle value, 127, of the dynamic range [0 255] of the pixels. Figure 5 plots the RMSE per frame for three video sequences, with high motion content. These plots further confirm the observations made in Figure 4. Weighted average with ∝ = 0.999, yields the lowest RMSE for most of the frames. At the start and during rapid scene changes, however, it rises to higher values, as discussed previously. The moving average performs poorly in all if the cases considered, except during a few frames in ‘Foreman’ and ‘Ice’ sequences. Thus, even for high-motion video sequences, the error in the centroid’s values, calculated through the weighted average (∝ = 0.999) is upper-bounded by the error for the moving average, with reference to the corresponding offline implementation.

RMSE values for the full test video sequences have been reproduced in Table 1. It can be observed from these values that the weighted average performs better than moving average on all the sequences on average. The former only occasionally performs poorly in sudden scene changes, and at the very start of the algorithm as observed in Figure 4 and Figure 5.

In conclusion, weighted average is a better choice than the moving average, not only due to its lower computational complexity, but also its better performance. Experimental evidence dictates that ∝ = 0.999 yields better results than the other choices. Decreasing this value leads to poorer overall performance. On the other hand, further increasing this value leads to poorer response on startup and high-motion-content frames. Moreover, increasing this value requires further precision in its representation which leads to subsequently more complex hardware.

### 3.2. Simulink Design Entry and High-Level Synthesis

**Figure 6:**
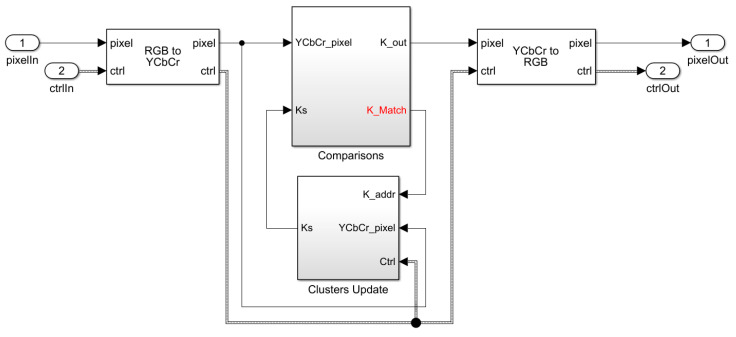
The Simulink model developed for the online K-Means clustering algorithm with Xilinx AXI-4 compliant standard pixel streaming interfaces.

The online K-Means algorithm has been implemented as a Simulink model to generate the corresponding Xilinx AXI4 streaming protocol-compatible IP core. The top-level module has been depicted in Figure 6.

**Figure 7:**
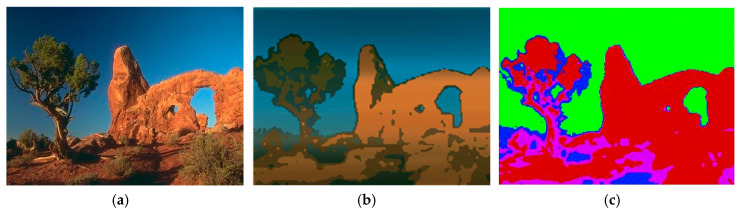
Image segmentation output options: (**a**) Input image [18]; (**b**) Output image with each pixel replaced by its corresponding cluster’s centroid value; (**c**) Output image with pseudo-colors to denote clustering.

As discussed earlier, the first operation performed on the pixel stream is the conversion from RGB to the YCbCr colorspace, in order to use only color components for segmentation. On the output side, the reverse transformation is necessary if the pixel values are replaced with their associated cluster values. The other option is to simply output the fixed colors that correspond to each identified cluster (pseudo-coloring), as shown in Figure 7. The former option gives a more pleasing output, but the latter may be more suitable for certain downstream tasks. For demonstration purposes, our hardware uses the former option, and this leads to slightly more resources being utilized by the YCbCr2RGB conversion. Both of these conversion modules are available in the Simulink Vision HDL toolbox.

**Figure 8:**
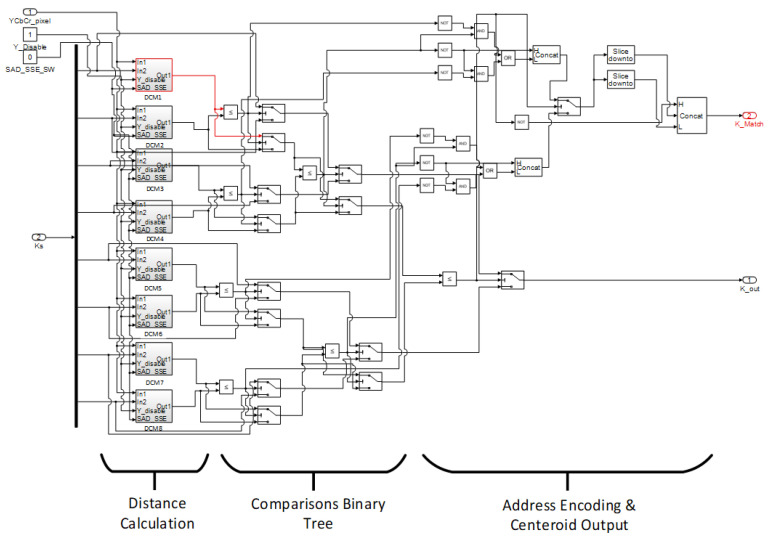
Comparison module to find the matching cluster’s centroid.

After the colorspace conversion, the color components, i.e., the *Cb* and *Cr* values of each pixel, are compared against the current centroids of each cluster (eight in our model) in the ‘Comparisons’ module. These centroids are initialized at random to ensure the proper operation of the K-Means clustering, as discussed in the literature. The “Comparisons” module outputs the classification value of the current pixel (Figure 8), as well as the address of the matched cluster for updating its centroid in the “Clusters Update” module. The updated module uses Equation (3) to output the new centroids for the next cycle. These two modules are elaborated below.

#### 3.2.1. Comparisons Module

The comparisons module takes in pixel data in the YCbCr format, and compares it with the corresponding centroids of eight clusters in the first stage. For this purpose, eight “Distance Calculation Modules” (DCM) are employed. These DCMs have the option to use either the L1 or L2-norm as the heuristic for a match, using “SAD_SSE_SW” switch. They can also include or exclude the ‘Y’ (luminance) component, while finding the best match between current pixel and the corresponding centroid through “Y_Disable” switch. The second stage is a binary tree of the comparators and multiplexers which successively propagates the centroid with minimum distance forward. Finally, the centroid of the best matching cluster and its 3-bit encoded address is output based on the logical outcome of each comparator.

#### 3.2.2. Clusters Update Module

**Figure 9:**
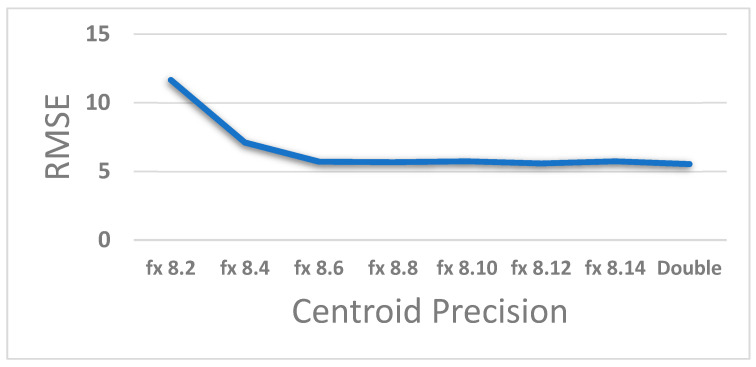
Effect of the fixed-point arithmetic on result accuracy.

The centroids of eight clusters are updated using the output from ‘Comparisons Module’ and the current pixel. The centroid values are stored in registers as fixed-point values using a word size of 18 bits with eight fractional bits. The precision for fractional bits has been decided, based on the accuracy loss behavior that is depicted in Figure 9. RMSE for test video sequences were gathered for different bit precisions. It was observed that the RMSE error metric shows a sharp rise when the fractional bits are reduced below 6. On the other hand, the allocation of up to 14 bits yields a performance that is at par with the double-precision floating point software implementation. Thus, eight fractional bits seems to be a reasonable choice. Ten further bits were allocated for the sign and magnitude, with a 1-bit margin for overflows. The centroids are initialized to random values around 127 at the start, as used in the experimental setup described in Section 3.1.

The update method (Equation (3)) has been implemented as a user-defined function module with the option to initialize the centroids at startup, using the ‘vStart’ signal that is available from AXI4 streaming bus. All other components have been implemented by using Simulink intrinsic modules that support direct synthesis. Hence, the entire design framework is highly flexible, with support for functionality testing by using Simulink media interfaces. Moreover, fixed-point hardware implementation can be compared against the corresponding full-precision software model.

**Figure 10:**
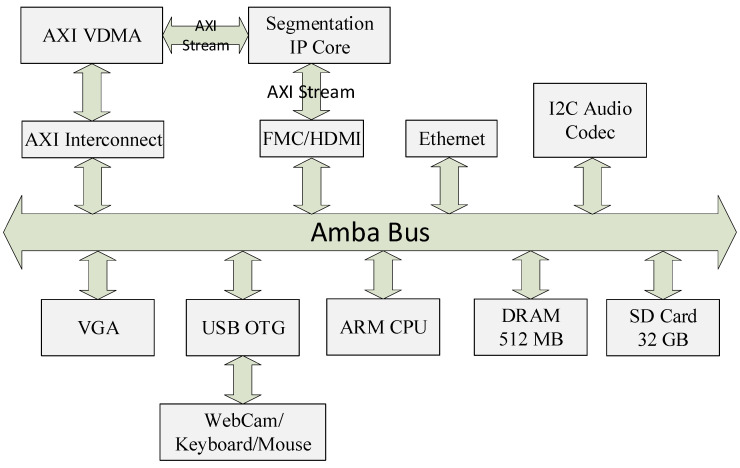
Hardware-software co-design architecture.

Finally, the Simulink HDL Coder is invoked to convert the hardware model into AXI4 streaming bus compliant IP core in the form of HDL sources. To test the functionality of this IP core in a practical environment, a Hardware–Software co-design (HW-SW) has been setup on a Xilinx Zedboard, which houses Xilinx Zynq-7000 AP SoC XC7Z020-CLG484 FPGA running at 100 MHz. The hardware portion has been implemented in the Xilinx Vivado tool, with all the peripherals, as well as the segmentation IP core connected across a single bus, as shown in Figure 10. The software environment is based on Xillinux, an operating system based on Ubuntu for ARM. The application to test the IP core functionality makes use of OpenCV computer vision library as well. This setup ensures that three different sources of video streams can be used to feed the developed IP core, i.e., the High Definition Multimedia Interface (HDMI) on the Zedboard, USB webcam or the stored files on the flash memory card decoded through software library. For the former two sources, the AXI Video DMA core accesses a dedicated section of Random Access Memory (RAM) to read/write input/output frames.

**Figure 11:**
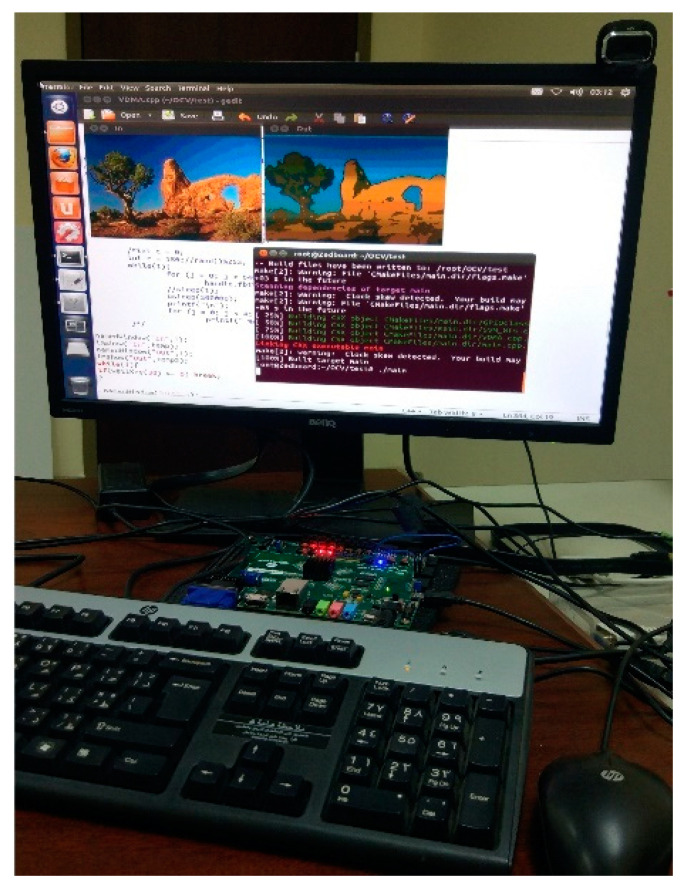
Hardware–software co-design implemented on the Zedboard FPGA platform.

Figure 11 shows an operating scenario where an image that is stored on the flash memory card is written to RAM in software. The segmentation IP core reads (via DMA) this image, processes it and then writes the output image (via DMA) to a different RAM segment. The software subsequently displays the output via OpenCV library functions. The continuous video stream from a USB webcam can be used as input in the similar fashion. For HDMI/FMC input/output, however, video capture and display devices need to be connected to the respective peripheral channel. For experimentation, the input/output frame size has been fixed at VGA resolution (640 × 480). The entire framework, including the Simulink models, the Vivado project files for the HW-SW co-design, and software routines are available for download as open-source code, to facilitate researchers and practitioners.

## 4. Results

The proposed IP core for image segmentation, using the online K-Means algorithm, has been synthesized, along with the entire HW-SW co-design, using Vivado 2016. The synthesis results have been reproduced in Table 2, and compared with those for similar structures reported in the literature.

Hussain’s hardware [25] for bioinformatics applications also uses fixed eight clusters, but it does not include the logic resources that are utilized by the interfaces in their final report. This design also does not include the colorspace conversion modules. Thus, in comparison, our design delivers more functionality for similar Look Up Table (LUT) resource consumption without utilizing any Block Random Access Memory (BRAM) parts. Their design is heavily parallelized, and it runs at 126 MHz. As a result, many more slice registers are consumed by the circuit. Furthermore, it requires on-chip BRAM, as well as the external main memory, for complete operation. Similarly, Kutty’s architecture [26] consumes a comparable number of logic resources, but even more registers and BRAM resources. This design also achieves a high operating frequency of 400 MHz by heavily pipelining the circuit. However, both of these designs require the external RAM for the cluster update feedback loop, as discussed in Section 2. Thus, achieving higher clock rates for the hardware through pipelining without the loop is meaningless, since the overall operation is much slower, due to the required accesses to the main memory. This fact has been recognized by Raghavan et al. [27] as well, who have described another hardware architecture for big-data applications. Cahnilho et al. [28] have only reported the hardware resource utilization for the comparisons module, and not for the full operation. Moreover, their design requires software intervention which prohibits its inclusion in an image processing pipeline. Li’s design [29] is based on a map-reduce technique, which may be suitable for big-data applications, but not for real-time image segmentation, since it requires an exorbitant amount of logic and Digital Signal Processing (DSP) resources for its implementation.

Table 2 also gives the breakup of the logic resource utilization and the estimated dynamic power consumption for the different constituent components in the proposed design. These values have been noted from the Vivado power estimation tool after a place-and-route task for the FPGA bit-stream generation. As expected, the clusters update module consumes the most resources, due to the presence of the fixed-point arithmetic implementation using Equation (3), and the associated registers. It also consumes the most dynamic power, i.e., 72 mW, due to these clocked registers. It should be noted, however, that these estimated power numbers have limited accuracy, and their absolute values are likely to be very different in practical scenarios. It should be noted that the colorspace conversion modules take up to 21% of the share of the slice LUTs, and almost 40% of the registers. These modules are synthesized via the built-in Simulink Vision HDL toolbox blocks.

In conclusion, the proposed hardware design is very well suited for real-time image segmentation, since it requires minimal logic resources, and it does not depend on the external memory for complete operation. As described earlier, and as is evident from Figure 10, the proposed design can be readily inserted into any generic image processing pipeline as a stand-alone IP core. Despite using high-level synthesis tool for its development, the developed core is efficient both in terms of resource utilization, speed and power consumption. The final synthesized core is able to run at 55 MHz, which translates to 59.7 fps and 26.5 fps for HD (1280 × 720) and Full HD (1920 × 1080) video resolutions respectively while consuming only little power (≈86 mW). To accommodate this lower clock, the AXI interface runs off a slower clock instead of the default 100 MHz system-wide clock. It may be reiterated that the designs reported earlier in the literature do not use the immediate feedback loop in their calculation, and hence, their mentioned speeds are not representative of the full-operation conditions. The low values of estimated power consumption further affirm the suitability of the developed IP core for low-power image processing pipelines.

In this paper, a fixed number of clusters, i.e., eight, was used to illustrate the design principle with weighted average in place of moving average. The extension to a larger number of clusters in powers of two is straightforward, given the modular nature of the design shown in Figure 8 (the comparisons module). The developed Simulink framework for the online K-Means clustering algorithm can be extended to include the EM and GMM algorithms, with minimal effort in the future. For this purpose, the online calculation of variance needs to be added, along with modifications to the distance calculation modules.

## Figures and Tables

**Table 1:** A comparison of online K-Means algorithms in terms of the average error in centroid values from the offline approach.

Video Sequence	Resolution	Number of Frames	RMSE	
			Moving Average	Weighted Average ∝ = 0.999
Akiyo	352 × 288	300	11.82	7.14
Container	352 × 288	300	7.51	7.13
Foreman	352 × 288	300	9.04	6.44
Carphone	352 × 288	382	6.62	5.77
Claire	176 × 144	494	9.56	6.75
Hall	352 × 288	300	5.96	4.21
Highway	352 × 288	2000	7.26	4.61
Soccer	352 × 288	150	8.06	4.71
Ice	352 × 288	240	3.75	3.01
Tennis	352 × 288	150	8.63	6.83

**Table 2:** FPGA synthesis results.

Design		FPGA	Slice LUT	Slice Registers	BRAM	DSP	Dynamic Power
Hussain [25]		Xilinx Virtex-IV	2208	3022	90 Kb	-	-
Kutty [26]		Xilinx Virtex-VI	2110	8011	288 Kb	112	-
Raghavan [27]		Xilinx Virtex-6	6916	14,132	-	88	-
Cahnilho [28]		Xilinx Zynq-7000	1583	1016	36 Kb	7	-
Li [29]		Xilinx Zynq-7000	178,185	208,152	5742 Kb	412	-
Proposed	Full IP	Xilinx Zynq-7000	3402	2443	0	62	86 mW
	AXI		458	538	0	0	4 mW
	CSC		719	1014	0	14	7 mW
	Clusters Update		1643	876	0	48	72 mW
	Comparisons		582	15	0	0	3 mW

## Supplementary Materials

The described hardware accelerator IP core and the relevant Simulink models, as well as the Vivado project for HW-SW co-design, are available for download at (https://sites.google.com/view/4mbilal/home/rnd/image-segmentation).
